# Body Surface Radiation Exposure in Interventional Echocardiographers During Structural Heart Disease Procedures

**DOI:** 10.1016/j.jacasi.2022.12.008

**Published:** 2023-03-28

**Authors:** Akihisa Kataoka, Takeshi Takata, Ayaka Yanagawa, Kento Kito, Masataka Arakawa, Ruri Ishibashi, Taiga Katayama, Miho Mitsui, Fukuko Nagura, Hideyuki Kawashima, Hirofumi Hioki, Yusuke Watanabe, Ken Kozuma, Jun’ichi Kotoku

**Affiliations:** a Division of Cardiology, Department of Internal Medicine, Teikyo University, Tokyo, Japan; b Advanced Comprehensive Research Organization, Teikyo University, Tokyo, Japan; c Department of Anesthesia, Teikyo University, Tokyo, Japan; d Department of Cardiovascular Medicine, Asahi General Hospital, Tokyo, Japan; e Graduate School of Medical Care and Technology, Teikyo University, Tokyo, Japan

**Keywords:** echocardiologist, female doctor, interventional echocardiographer, radiation exposure, structural heart disease, transesophageal echocardiography

## Abstract

**Background:**

The distribution of radiation exposure on the body surface of interventional echocardiographers during structural heart disease (SHD) procedures is unclear.

**Objectives:**

This study estimated and visualized radiation exposure on the body surface of interventional echocardiographers performing transesophageal echocardiography by computer simulations and real-life measurements of radiation exposure during SHD procedures.

**Methods:**

A Monte Carlo simulation was performed to clarify the absorbed dose distribution of radiation on the body surface of interventional echocardiographers. The real-life radiation exposure was measured during 79 consecutive procedures (44 transcatheter edge-to-edge repairs of the mitral valve and 35 transcatheter aortic valve replacements [TAVRs]).

**Results:**

The simulation demonstrated high-dose exposure areas (>20 μGy/h) in the right half of the body, especially the waist and lower body, in all fluoroscopic directions caused by scattered radiation from the bottom edge of the patient bed. High-dose exposure occurred when obtaining posterior-anterior and cusp-overlap views. The real-life exposure measurements were consistent with the simulation estimates: interventional echocardiographers were more exposed to radiation at their waist in transcatheter edge-to-edge repair than in TAVR procedures (median 0.334 μSv/mGy vs 0.053 μSv/mGy; *P <* 0.001) and in TAVR with self-expanding valves than in those with balloon-expandable valves (median 0.067 μSv/mGy vs 0.039 μSv/mGy; *P <* 0.01) when the posterior-anterior or the right anterior oblique angle fluoroscopic directions were used.

**Conclusions:**

During SHD procedures, the right waist and lower body of interventional echocardiographers were exposed to high radiation doses. Exposure dose varied between different C-arm projections. Interventional echocardiographers, especially young women, should be educated regarding radiation exposure during these procedures. (The development of radiation protection shield for catheter-based treatment of structural heart disease [for echocardiologists and anesthesiologists]; UMIN000046478)

Structural heart disease (SHD) is a new field in cardiovascular medicine. Echocardiologists play a key role in ensuring proper patient selection and in the technical success of SHD procedures via echocardiographic monitoring and guidance.^1^ However, risks of radiation exposure to echocardiologists who perform transesophageal echocardiography (TEE) in cardiac catheterization or hybrid cardiac surgical suites have been noted.^2^ Protective equipment such as mobile, lead-containing, acrylic sheets, and lead curtains are typically only used by catheter operators in cardiac catheterization suites.3,4 Interventional echocardiographers are exposed to a higher radiation dose than the first catheter operator in SHD procedures.^5^, ^6^, ^7^

More female doctors practice echocardiography than other invasive subspecialties in the U.S. and Japan.^8^^,^^9^ Radiation exposure in female health care workers should not exceed 20 mSv/y over 5 years for the lens of the eye, 500 mSv/y for the skin, and 1 mSv during the pregnancy period for the embryo/fetus.^3^^,^^4^^,^^10^^,^^11^ In addition, echocardiologists who are involved in transcatheter edge-to-edge repairs (TEERs) tend to be younger than other health care workers.^9^ Therefore, radiation exposure during SHD procedures is a major challenge for female echocardiologists, especially those in training or those who are beginning their careers, as these career phases often coincide with the childbearing years.^1^^,^^8^

Monitoring the exposure dose as accurately as possible is extremely important to avoid radiation-related injuries.^12^ The distribution of radiation exposure on the body surface of each interventional echocardiographer is still unclear. Therefore, this study estimated and assessed the radiation exposure on the body surface of interventional echocardiographers performing TEE in a hybrid cardiac surgical suite using a Monte Carlo simulation and measuring real-life radiation exposure during SHD procedures.

## Methods

### Monte Carlo simulation system

The fast dose estimation system for interventional radiology (FDEIR) Monte Carlo system was used to estimate the exposure dose.^12^ FDEIR simulates the radiation exposure dose in the diagnostic energy range using the Monte Carlo method. Previous studies have validated its accuracy using dosimeters and other Monte Carlo approaches.^12^, ^13^, ^14^ The simulation was conducted using a single Tesla P100 graphical processing unit (NVIDIA Corp) on a supercomputing system (SGI Rackable C2112-4GP3/C1102-GP8, Reedbush-L, Silicon Graphics International Corp) at the Information Technology Center of the University of Tokyo. This study simulated trillion incident photons with a 5-keV cutoff energy. The simulation suppressed electron transport, thereby accelerating the calculation.

### Calibration factor

FDEIR calculates the relative dose per number of incident photons instead of the absolute radiation dose. Therefore, a calibration factor that defines the number of photons per mAs was used to convert the simulated relative dose to an absolute dose using FDEIR and a radiophotoluminescence dosimeter (GD-352M, Chiyoda Technol Corp), based on a previously reported method.^12^ The geometry was set to obtain the calibration factor (Supplemental Figure 1). Two radiophotoluminescence dosimeters were placed in a water-equivalent phantom at the center of the field of view. The doses were measured under 12 conditions, changing the source-to-surface distance (60-70 cm) and depth (0-15 cm). All other conditions are listed in Supplemental Table 1. A voxelized geometry resembling the measurement geometry was constructed (Supplemental Figure 1) to simulate the dose under the same conditions as the measurements obtained during SHD procedures. The relationship between the simulated dose was approximated using a linear function with zero intercept.^12^ The slope of the line represented the calibration factor.

### Simulation of radiation exposure of interventional echocardiographers

**Figure 1:**
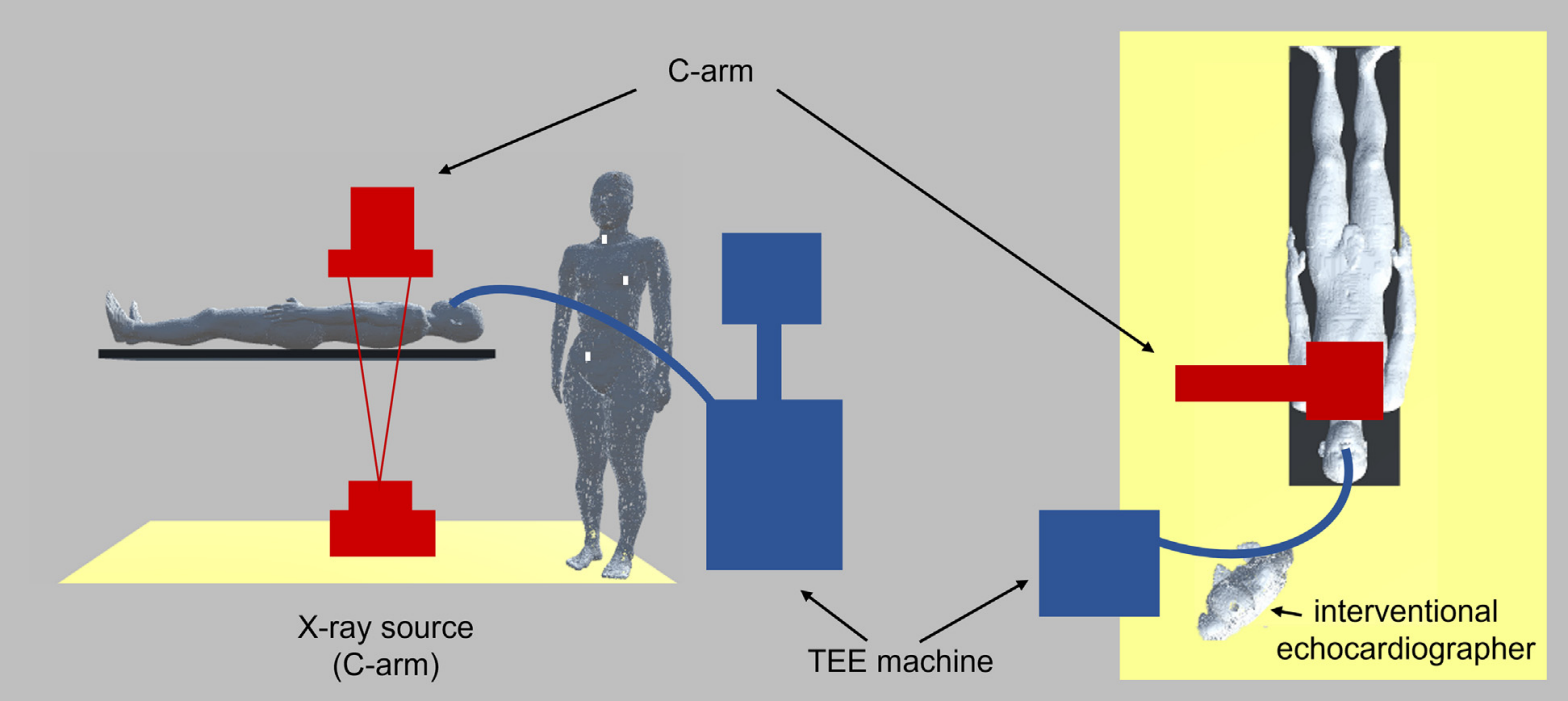
Clinical Study and Simulation Geometry in Hybrid Cardiac Surgery Suite The C-arm and transesophageal echocardiography (TEE) machine were not included in the simulation. The distance from the center of the x-ray source to the center of the interventional echocardiographer’s body was set at 112 cm in the simulation. Although it varies from case to case, it was approximately 110 to 120 cm in the clinical study. The measurement points for radiation exposure on the neck, chest, and waist used during the clinical study are represented by **white squares**. See Supplemental Figure 1 for more information.

To estimate the exposure dose of echocardiologists who perform continuous TEE monitoring and guidance, a voxelized geometry divided into mesh voxels (2 mm × 2 mm × 2 mm) resembling a hybrid cardiac surgery suite at our institution was constructed, using an under-table x-ray source, imaging table, air, concrete floor, whole-body male model as a patient, and whole-body female model as a physician (Figure 1). The interventional echocardiographer model reflected the actual posture of interventional echocardiographers.^15^ Mass density and compositions were assigned for the ocular lens, thyroid gland, ovary, bone, and skin, based on the International Commission on Radiological Protection publication 110.^16^ For simplicity, other tissues were treated as water or air.

The risk of radiation dermatitis during TEER of the mitral valve—where the x-ray beam used for cardiac fluoroscopy was aimed at the heart of the patient model—was determined in the simulation. These conditions were determined based on the procedure for TEER of the mitral valve at our institution using a fluoroscopic unit (Allura Xper FD20 X-ray system). The fluoroscopic conditions are presented in Supplemental Table 1.

### Clinical study to measure radiation exposure of interventional echocardiographers

**Figure 2:**
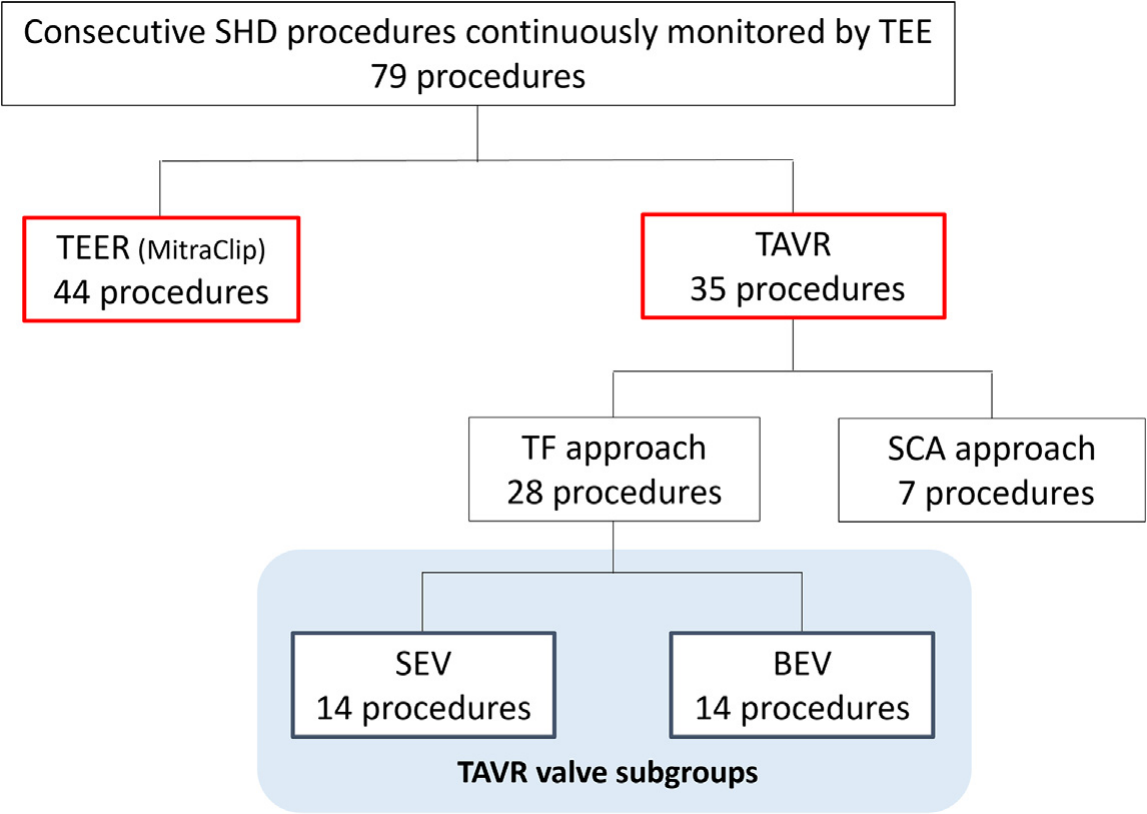
Clinical Study Flowchart A total of 79 consecutive structural heart disease (SHD) procedures (including 44 transcatheter edge-to-edge repairs [TEERs] and 35 transcatheter aortic valve replacements [TAVRs]) using continuous transesophageal echocardiography (TEE) were performed at our institution between April 1, 2021, and December 24, 2021. Of the 35 TAVR procedures, 7 were performed via the trans-subclavian artery (SCA) approach and were, therefore, excluded from the study. The remaining 28 TAVR procedures were performed via transfemoral (TF) approach and were included in the study. They were further grouped and analyzed according to the TAVR valve: 14 self-expanding valve (SEV) and 14 balloon-expandable valve (BEV) procedures.

A retrospective, observational, single-center study including 79 consecutive SHD procedures using continuous TEE was conducted. The SHD procedures included 44 TEERs of the mitral valve using the MitraClip device (Abbott Vascular) and 35 transcatheter aortic valve replacements (TAVRs) conducted between April 1, 2021, and December 24, 2021 (Figure 2). All procedures were performed by the 2 interventional attending cardiologists (H.H., Y.W.) (each with more than 15 years of experience) and 3 echocardiography fellows (K.K., M.A., R.I.) (each with over 4 years of experience), under the consistent guidance and supervision of an echocardiography attending cardiologist with 19 years of experience in clinical practice. The study protocol was developed in accordance with the 1975 Declaration of Helsinki and its later amendments and was approved by the Institutional Review Board of Teikyo University (approval number TEIRIN 20-178 and 21-100). All participants provided informed consent in written form. This trial was registered with the University Hospital Medical Information Network (UMIN000046478). A semiconductor personal radiation dosimeter (Hitachi, Ltd) was used to measure the radiation exposure dose at the surface of the interventional echocardiographer’s neck (right side), chest (left pocket location of the radiation protective clothing), and waist (right side). The dosimeters were attached to the outside of the radiation protective clothing, and no external shields or absorbing devices such as RADPAD® (Worldwide Innovations and Technologies Inc) were used, which represents the typical setup in the hybrid cardiac surgery suite at our institution.

### Statistical analysis

Continuous variables are presented as median (IQR). Pearson’s correlation coefficient was used to assess the degree of agreement of calibration factors for the Monte Carlo simulation. The differences between groups were tested using analysis of variance with the Scheffé post hoc test or Mann-Whitney test. All statistical analyses were performed using the MedCalc software version 19.1.3 (MedCalc Software). Statistical significance was set at *P* < 0.05.

## Results

### Calibration factor

The measured and simulated doses with the least-squares fitting straight line for computing the calibration factor are shown in Supplemental Figure 2. The linear regression equation describing their relationship was y=1.10x. The coefficient of determination (the square of Pearson’s correlation coefficient [r = 0.995]) was 0.991, suggesting that the measured and simulated doses were proportional, and that linear regression was appropriate.

### Monte Carlo simulation

**Figure 3:**
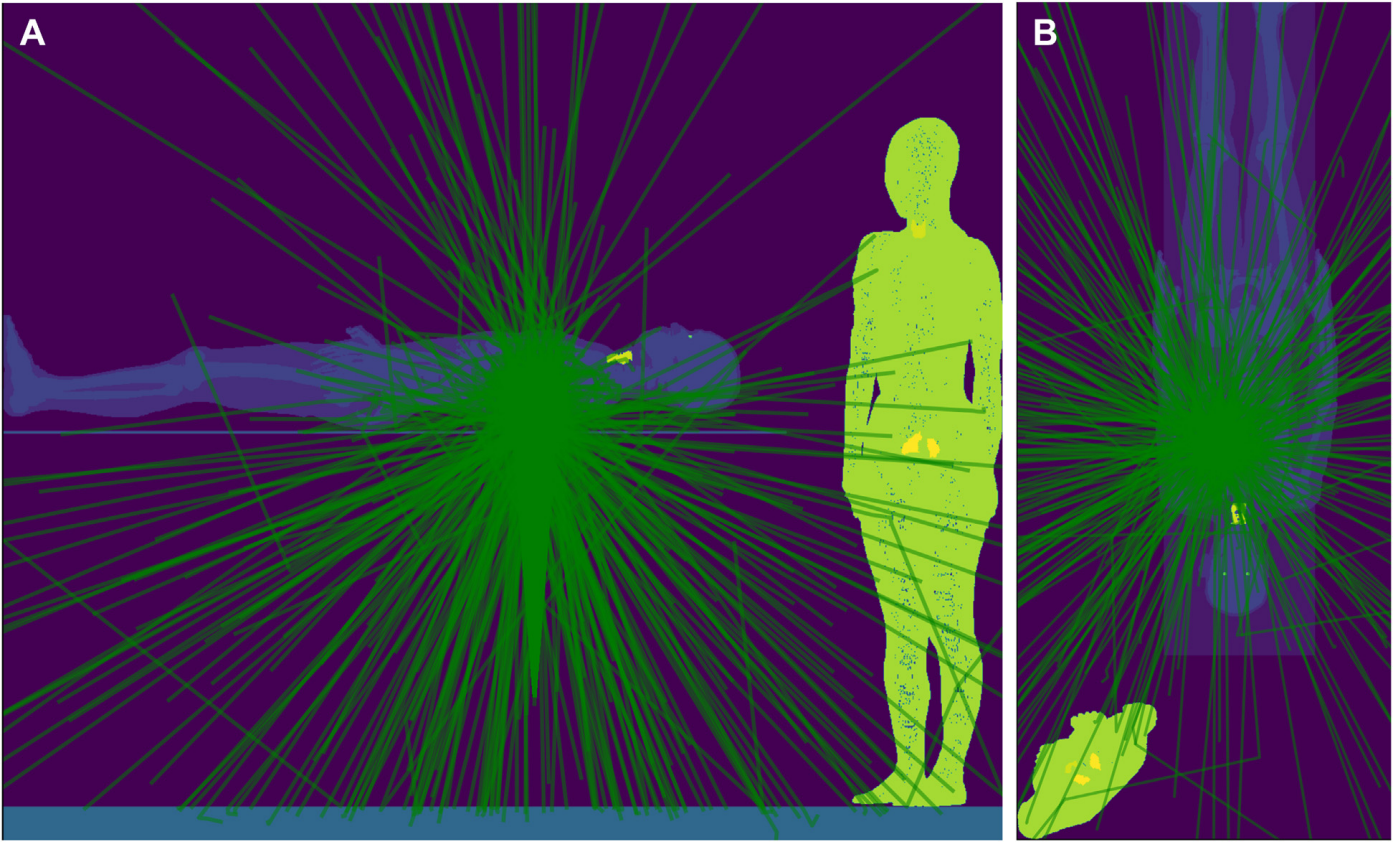
X-Ray Photon Trajectory During the Simulation Track analysis confirmed that the x-ray photons followed a plausible track. **(A)** Side view, **(B)** top view. Photons enter from the posterior-anterior direction, with Compton scattering toward the lower end of the patient bed, exposing mainly the lower body of the interventional echocardiographer performing transesophageal echocardiography. The **green lines** are the scattered photon trajectories calculated by Monte Carlo simulation.

The dose rate distribution map of the interventional echocardiographer’s body surface is shown in Central Illustration A. High-dose exposure (>20 μGy/h) was observed in the right half of the body, especially the waist and lower body, during all fluoroscopic views. However, the distribution of the high-dose area varied between views. A high-dose area appeared in the posterior-anterior (PA) and cusp-overlap views. In the perpendicular view, the high-dose region was remarkably narrow. Central Illustration B demonstrates the absorbed dose rates for the skin and left and right lenses, which were calculated at the white (for right) and black (for left) boxes (2.0 cm × 1.0 cm) shown on the interventional echocardiographer’s body model in Central Illustration A. The cusp-overlap had the highest, PA had the second highest, and perpendicular view had the lowest absorbed dose rates at all positions (all *P <* 0.05). All right-sided measurement locations tended to have higher absorbed dose rates than left-sided measurement locations. The x-ray photon trajectory follows a plausible path (Figure 3).

**Central Illustration:**
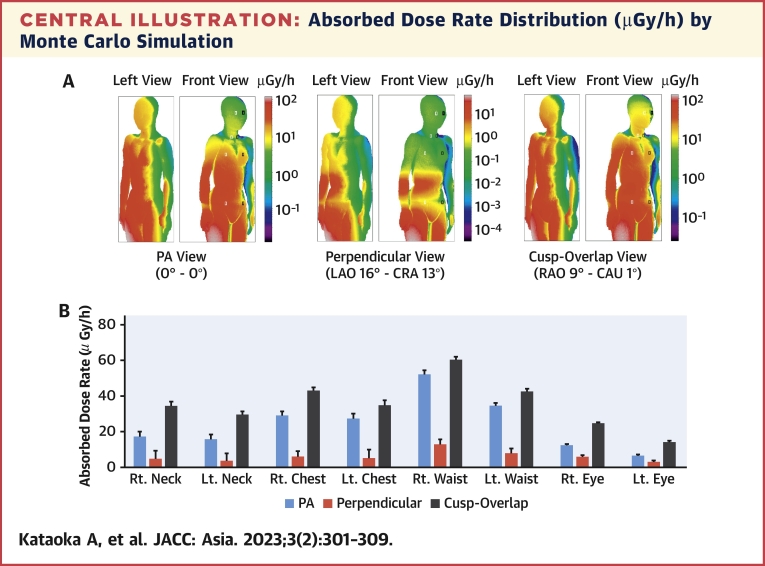
Absorbed Dose Rate Distribution (μGy/h) by Monte Carlo Simulation **(A)** The absorbed dose rate distribution of the body surface of the interventional echocardiographer is shown. **(Left)** The left view of the interventional echocardiographer model, **(right)** the front view of each fluoroscopy view. The high-dose exposure dose areas are expressed in **red** (>20 μGy/h). The **white (right)** and **black (left) boxes** (2.0 cm × 1.0 cm) were used to calculate the absorbed dose rates. **(B)** The absorbed dose rates during the posterior-anterior, perpendicular, and cusp-overlap views are shown for the skin and right and left eye lenses. The **error bars** represent statistical error (+1σ). The cusp-overlap had the highest, PA the second-highest, and the perpendicular view had the lowest absorbed dose rates at all positions (all *P <* 0.05). CAU = caudal; CRA = cranial; LAO = left anterior oblique; Lt. = left; PA = posterior-anterior; RAO = right anterior oblique; Rt. = right.

### Clinical study measuring radiation exposure

Overall, greater radiation exposure was found at the waist than at the chest (median: 11.5 μSv vs 2.0 μSv; *P <* 0.05) (Table 1). The overall group had higher radiation exposure at the waist (*P <* 0.05) than at the chest. Differences in the radiation exposure per cumulative air kerma (CAK) in the neck, chest, and waist were observed between the overall and TEER groups (both at *P <* 0.001). The overall and TEER groups had higher radiation exposure per CAK at the neck (both at *P <* 0.05) and waist (both at *P <* 0.01) than at the chest. The TAVR group had a higher CAK (253.0 mGy vs 26.2 mGy; *P <* 0.001) and radiation exposure at the chest (2.0 μSv vs 1.0 μSv; *P <* 0.05) than the TEER group. The TEER group had higher radiation exposure per CAK at the neck (0.222 μSv/mGy vs 0.027 μSv/mGy; *P <* 0.001) and waist (0.334 μSv/mGy vs 0.053 μSv/mGy; *P <* 0.001) than the TAVR group.

**Table 1:** Procedural and Radiation Exposure Dose Data for Each Procedural Group in the Clinical Study

	Overall (N = 79)		TEER (n = 44)		TAVR (n = 35)		*P* Value
Fluoroscopy time, min	15.7 (11.6-22.6)		15.5 (11.2-22.8)		15.6 (11.7-17.6)		0.365
Procedure time, min	43.0 (33.0-56.0)		43.0 (31.0-58.5)		44.0 (34.5-56.0)		0.703
CAKD, mGy	133.2 (25.1-249.8)		26.2 (17.8-60.2)		253.0 (208.8-389.3)		<0.001
Neck, μSv	6.5 (4.0-15.5)	*P* = 0.012	6.0 (4.0-9.5)	*P* = 0.016	8.0 (4.0-16.0)	*P* = 0.575	0.147
Chest, μSv	2.0 (0-5.5)		1.0 (0-4.0)		2.0 (1.0-8.5)		0.044
Waist, μSv	11.5 (7.0-25.5)^a^		10.5 (6.0-15.0)		13.0 (7.2-31.0)		0.075
Neck/CAK, μSv/mGy	0.081 (0.027-0.232)^a^	*P <* 0.001	0.222 (0.107-0.283)^a^	*P <* 0.001	0.027 (0.017-0.050)	*P* = 0.250	<0.001
Chest/CAK, μSv/mGy	0.012 (0.000-0.053)		0.024 (0.000-0.095)		0.008 (0.003-0.027)		0.395
Waist/CAK, μSv/mGy	0.184 (0.055-0.341)^b^		0.334 (0.235-0.469)^b^		0.053 (0.031-0.074)		<0.001

Values are median (IQR).

CAK = cumulative air kerma; TAVR = transcatheter aortic valve replacement; TEER = transcatheter edge-to-edge repair.

a *P <* 0.05 vs chest.

b *P <* 0.01 vs chest.

### TAVR valve subgroups analysis

Of the 35 TAVR procedures, 28 were via transfemoral approach (14 self-expanding valves [SEVs], Evolut family, Medtronic; and 14 balloon-expandable valves [BEVs], SAPIEN 3, Edwards Lifesciences) and 7 were via trans-subclavian approach (Table 2). When the transfemoral approach was used, the SEV group had a longer irradiation time (median 17.4 min vs 13.1 min; *P <* 0.05) and a higher radiation exposure per CAK at the waist (median 0.067 μSv/mGy vs 0.039 μSv/mGy; *P <* 0.01) than the BEV group.

**Table 2:** Procedural and Radiation Exposure Dose Data for the Type of Transcatheter Heart Valve in TAVR

	SEV (n = 14)	BEV (n = 14)	*P* Value
Fluoroscopy time, min	17.4 (14.8-20.9)	13.1 (8.1-16.1)	0.016
Procedure time, min	44.0 (37.0-49.0)	36.5 (31.0-45.0)	0.240
CAK, mGy	271.7 (219.8-324.9)	260.5 (215.3-414.9)	0.926
Neck, μSv	9.5 (4.0-17.0)	7.5 (4.0-16.0)	0.889
Chest, μSv	1.0 (1.0-7.0)	2.5 (0.0-7.0)	0.779
Waist, μSv	20.0 (10.0-46.0)	10.0 (6.0-19.0)	0.072
Neck/CAK, μSv/mGy	0.031 (0.016-0.054)	0.025 (0.016-0.040)	0.747
Chest/CAK, μSv/mGy	0.004 (0.003-0.016)	0.008 (0.000-0.025)	0.926
Waist/CAK, μSv/mGy	0.067 (0.052-0.125)	0.039 (0.027-0.052)	0.005

Values are median (IQR).

BEV = balloon-expandable valve; CAK = cumulative air kerma; SEV = self-expanding valve.

## Discussion

To our knowledge, this is the first report estimating and visualizing radiation exposure on the body surface of interventional echocardiographers, corresponding to at-risk organs during SHD procedures, conducted in a hybrid cardiac surgical suite using a simulation and real-life measurements. The Monte Carlo simulation calculations in this study were consistent with those of a previous study.^12^ In addition, the clinical study results were consistent with the Monte Carlo simulation, as the high-dose exposure area was mainly observed on the right side of the interventional echocardiographer, especially in the waist and lower body. The distribution of the high-dose exposure area differed among different fluoroscopy views (C-arm direction), and the PA and cusp-overlap views had higher dose absorption overall. Therefore, the real-life radiation exposure of interventional echocardiographers differed based on the procedure.

The simulation demonstrated high absorbed dose rates in the waist and lower body, which were similar to the catheter operator’s pelvic radiation exposure during percutaneous coronary procedures at the catheterization suite; therefore, the ovary is at risk for radiation exposure.^17^ This exposure pattern is caused by the radiation scattering at the bottom edge of the patient bed. The real-life measured exposure in the waist and lower body was significantly higher than that in the chest, indicating that interventional echocardiographers, especially young female doctors in their childbearing years, must be considered at risk of high-dose exposure. In this study, the interventional echocardiographers used an echo screen during the procedures, placing them at an oblique right anterior position with respect to the C-arm, which was the radiation source. Therefore, the right side of the body was directly exposed to radiation, and the left side was shadowed, resulting in a higher exposure dose on the right side. During daily catheter intervention, radiation exposure is typically monitored at the left breast pocket of the radiation protective clothing, which is where the exposure dose of the chest was measured in this study. However, based on the simulation results, monitoring the radiation at this location may underestimate the exposure of the interventional echocardiographer’s right side, which may contribute to the fact that the radiation dose measured at the chest was lower than that at other locations in the clinical study.

Radiation-related skin injury is one of the hazards in various interventional radiology and TAVR procedures.^18^^,^^19^ Based on the simulation results, interventional echocardiographers may not reach the radiation dose limit for the skin, which is 500 mSv/y, and therefore their risk of radiation dermatitis and cancer are low.^20^ In contrast, eye lenses, which are also at-risk organs,^4^^,^^10^^,^^11^^,^^16^ are still at risk for developing cataracts, particularly the right eye lens. The equivalent radiation dose limit of eye lenses was drastically reduced from 150 mSv/y to 100 mSv per 5 years and 50 mSv/y in Japan, based on the recommendation of the International Commission on Radiological Protection Publication 118.^3^^,^^11^ The use of protective eyewear to reduce lens exposure is therefore recommended. However, the interventional echocardiographer was at an oblique right anterior position during TEE monitoring, and the radiation could enter the eye through the gap in the eyewear. Thus, to reduce lens exposure in this situation, appropriate protective eyewear with side protection that securely fits on the face should be used.^21^

The simulation demonstrated that absorbed dose rates were higher in the PA and cusp-overlap views, especially on the right side. The TEER procedure, in which the PA view is the basic fluoroscopic view, resulted in more radiation exposure than the TAVR procedure. Although the TEER of the mitral valve is typically performed using more TEE than fluoroscopy guidance, interventional echocardiographers should be aware of radiation exposure during the procedure. More radiation exposure at the waist was observed in the SEV group than in the BEV group. This may be because the cusp-overlap view, in which the right anterior oblique angle is used, is currently recommended for SEV implantation during the TAVR procedure.^22^ A previous study reported that the right anterior oblique angle C-arm projections predominantly increase the radiation exposure dose of interventional echocardiographers.^5^ Furthermore, because SEV requires a longer time to achieve full expansion than BEVs during deployment, the irradiation time was significantly longer than that of the BEV group. Therefore, interventional echocardiographers face longer exposure times and higher exposure rates at their right waist and lower body during transfemoral TAVR with a SEV.

The basic tools of occupational radiological protection are time, distance, and shielding.^16^ However, it is difficult for the interventional echocardiographer to reduce the exposure time and maintain distance during SHD procedures, because of the need for continuous TEE monitoring and guidance. Therefore, an additional ceiling-suspended lead shield, which dramatically reduces the exposure of the interventional echocardiographer,^2^^,^^5^^,^^23^ should be used during SHD procedures, especially for young, female interventional echocardiographers. Occupational doses can be reduced with the appropriate use of ceiling-suspended lead screens and protective lead curtains that are suspended from the sides of the procedure table.^16^ However, this setup is not easy to accomplish in hybrid cardiac surgical suites. Several institutions use ceiling-traveling C-arms during SHD procedures conducted in hybrid cardiac surgery suites. However, rails that allow the C-arm to move must be installed on the ceiling, thus limiting the location of the C-arm. In addition, a high-efficiency particulate air filter must be installed on the ceiling without interfering with the rails of the C-arm. Shadowless lights are also needed in the hybrid cardiac surgery suite. Hence, because of all the equipment needed on the ceiling, many institutions cannot install ceiling-suspended protective panels.^3^ In addition, lead curtain rubber shields that protect against exposure from scattered radiation can often not be hung from surgical beds based on product specifications.^3^ Therefore, a freestanding floor-mounted protective board should be used. Although a prototype for use during SHD procedures has been reported,^24^ there are no available products that do not interfere with the TEE monitoring and guidance equipment. Such products should be developed as soon as possible.

Radiation exposure during SHD procedures is not limited to interventional echocardiographers and catheter operators. The SHD procedure is performed under general or local anesthesia.^25^ Therefore, anesthesiologists who work near the location of the echocardiologist may also be exposed, as noted in a previous study.^26^ As more young women have become anesthesiologists in recent years,^27^ their radiation exposure during SHD procedures should also be investigated.

### Study limitations

First, the body surface dose estimation was conducted using a simulation with FDEIR. This simulation did not provide a detailed dose distribution for at-risk internal organs. In addition, interventional echocardiographers wore radiation-protective clothing, which was not included in the simulation. Second, it was reported that a high patient body mass index was the strongest predictor for radiation exposure of operators who performed diagnostic cardiac angiography.^28^ However, the patients’ habitus were not taken into account in this clinical study. Third, this study was a single-center observational and—in part—a simulation study using the cardiac surgery hybrid suite setup of our institution. The effects of scattering may vary depending on the equipment placement, which differs between institutions. In addition, at some institutions, TEER procedures for the mitral valve are conducted in angiography rooms, which may also affect radiation exposure. Fourth, although continuous TEE monitoring and guidance under general anesthesia was assumed in this study, intermittent transthoracic echocardiographic monitoring under local anesthesia is often used during TAVR procedures. In this study, TAVR procedures that used transthoracic echocardiographic monitoring had different exposure conditions than other procedures. Fifth, all of the dosimeters were attached to the outside of the protective clothing. Therefore, this study dramatically represented an overestimation of the radiation exposure at the body surface of an interventional echocardiographer, as it would be if the professional did not wear any external radiation protection clothing covering those areas. Finally, the real-life radiation doses to the right and left eye lenses were not measured in this study. Further investigations of these procedures with larger study cohorts and simulations based on various conditions will be needed to compensate for these limitations.

## Conclusions

During the SHD procedure, the interventional echocardiographer performing the TEE was exposed to high doses of radiation at the right half of their body, especially the waist and lower body, caused by scattered radiation from the bottom edge of the patient bed. The regional dose varied during different C-arm projections. These results suggest that education and appropriate shielding regarding radiation protection during SHD procedures is warranted for echocardiologists, especially young female doctors. In the future, the focus should be on developing shields that do not interfere with TEE equipment during SHD procedures. Considering the limitations of our study, further larger studies should be conducted, as well as additional simulations of various conditions.

Perspectives **COMPETENCY IN MEDICAL KNOWLEDGE:** During the SHD procedure, the interventional echocardiographers performing TEE were exposed to high-dose radiation at the right half of their body, especially the waist and lower body, caused by scattered radiation from the bottom edge of the patient bed. The regional dose varied during different C-arm projections. Interventional echocardiographers, especially young female doctors, should be made aware of radiation exposure during procedures where posterior-anterior and right anterior oblique fluoroscopic views are obtained.

**TRANSLATIONAL OUTLOOK:** Simulation-based and clinical assessments of the radiation exposure of anesthesiologists during SHD procedures are necessary. Novel shields such as protective boards that do not interfere with TEE equipment should be developed for use during SHD procedures.

## Funding Support and Author Disclosures

This study was supported by the Terumo Life Science Foundation, Japan 2020 R&D subsidies, the Abbott Medical Japan LLC scholarship fund, and the Boston Scientific Co scholarship fund. This work was also supported by the Japan Society for the Promotion of Science (21K07656 and 22H05108) and the Japan Science and Technology Agency ERATO Grant (JPMJER2102). The financial support was used to fund the semiconductor personal radiation dosimeters used in this study and for the Monte Carlo calculation. Drs Kataoka and Watanabe received remuneration from Abbott Medical Japan as proctors for MitraClip. All other authors have reported that they have no relationships relevant to the contents of this paper to disclose.
