# Supplementary material for: Body Surface Radiation Exposure in Interventional Echocardiographers During Structural Heart Disease Procedures
Source: JACC Asia. 2023 Mar 28;3(2):301–9. doi: 10.1016/j.jacasi.2022.12.008 (PMC10167512; doi:10.1016/j.jacasi.2022.12.008)

**Supplemental Table 1.** Fluoroscopic conditions used at our institution

| Tube voltage | 80 kV |
| --- | --- |
| mA | 3.0 |
| Fps | 3.75 |
| Field of view | 156.5 mm × 156.5 mm |
| Source to surface distance (in the PA direction) | 60 cm |
| Source to image-receptor distance | 100 cm |
| Filter | 2.5 mmAl + 0.4 mmCu + 1.0 mmAl |

Abbreviation: PA; posterior-anterior

**Supplemental Figure 1. Geometric arrangement for obtaining the scaling factor**

Radiophotoluminescence dosimeters were placed between rubber water-equivalent phantoms to obtain the measurements.

Abbreviation: SSD, source-to-surface


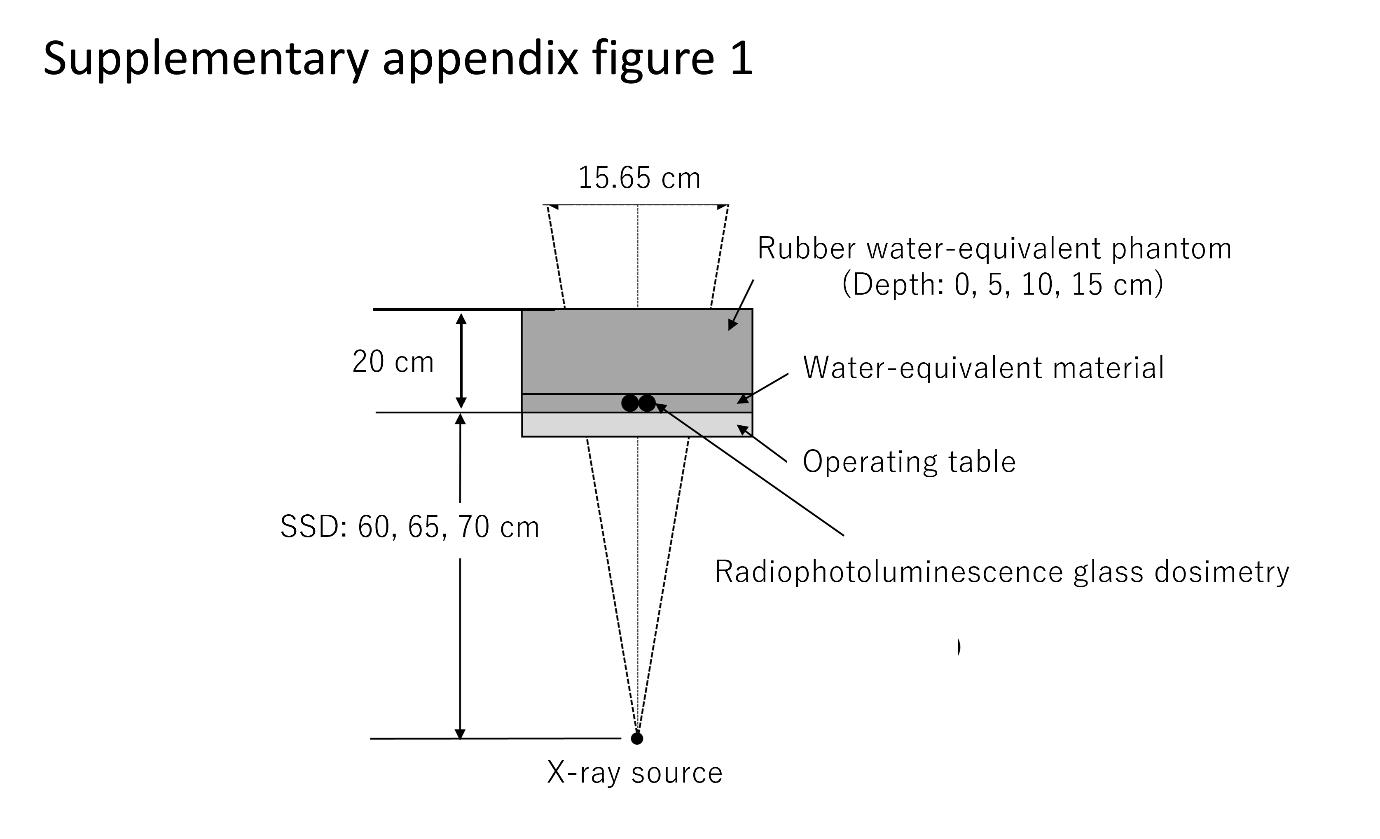


**Supplemental Figure 2. Measured and simulated dose with the least-squares fitting straight line for computing the calibration factor**
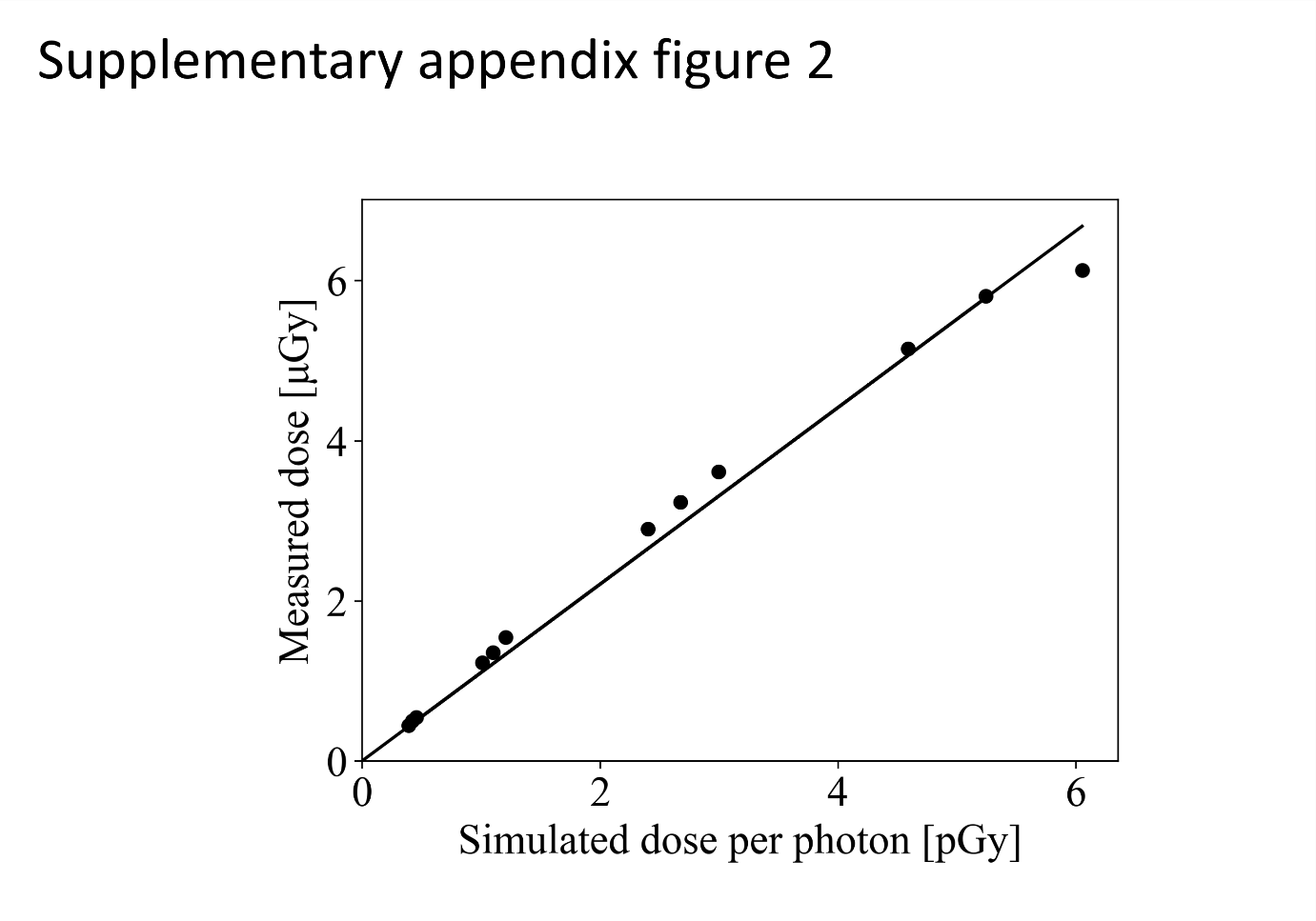

Supplement: Supplemental Table 1 and Figures 1 and 2 [file mmc1.docx]
